# Unique roles of rare variants in the genetics of complex diseases in humans

**DOI:** 10.1038/s10038-020-00845-2

**Published:** 2020-09-18

**Authors:** Yukihide Momozawa, Keijiro Mizukami

**Affiliations:** 1 Laboratory for Genotyping Development, RIKEN Center for Integrative Medical Sciences, Kanagawa, Japan; 2 Laboratory for Molecular Science for Drug Discovery, Graduate School of Medical Life Science, Yokohama City University, Kanagawa, Japan

**Keywords:** Disease genetics, Immunological disorders, Cancer genetics

## Abstract

Genome-wide association studies have identified >10,000 genetic variants associated with various phenotypes and diseases. Although the majority are common variants, rare variants with >0.1% of minor allele frequency have been investigated by imputation and using disease-specific custom SNP arrays. Rare variants sequencing analysis mainly revealed have played unique roles in the genetics of complex diseases in humans due to their distinctive features, in contrast to common variants. Unique roles are hypothesis-free evidence for gene causality, a precise target of functional analysis for understanding disease mechanisms, a new favorable target for drug development, and a genetic marker with high disease risk for personalized medicine. As whole-genome sequencing continues to identify more rare variants, the roles associated with rare variants will also increase. However, a better estimation of the functional impact of rare variants across whole genome is needed to enhance their contribution to improvements in human health.

## Introduction

Over the last 15 years, genome-wide association studies (GWAS) have identified >10,000 (common in most cases) genetic variants associated with various diseases and phenotypes [1, 2]. Although the causal variants and genes to directly increase or decrease different disease risks and phenotypes remain unknown in most GWAS loci, positional (and functional) candidate genes in GWAS loci, as well as integrated analysis with other functional datasets, have elucidated novel biological pathways involved in a target disease. Polygenic risk scores per individual successfully identified high-risk individuals in a part of complex diseases [3]. In these GWAS, rare variants with lower minor allele frequency (MAF) (e.g., <1%) have been examined using a customized SNP array and imputation. Customized SNP arrays can be used to focus on the rare variants in the genes of interest for different types of diseases, namely Immunochip array for major autoimmune and inflammatory diseases [4], Metabochip array for metabolic, cardiovascular, and anthropometric traits [5], and iCOGS array for hormone-related cancers [6]. Imputation using reference panels has been used to infer the genotypes of rare variants not directly genotyped by SNP arrays [7]. However, it is not able to analyze all rare variants. Custom arrays have been used to focus on rare variants in genes of interest, which are previously identified in European populations [4]. Imputation has a limited accuracy, especially for rarer variants with <0.1% of MAF. This accuracy depends on the size of the reference panel and genetic background and is known to be lower in non-European populations [8].

**Fig. 1:**
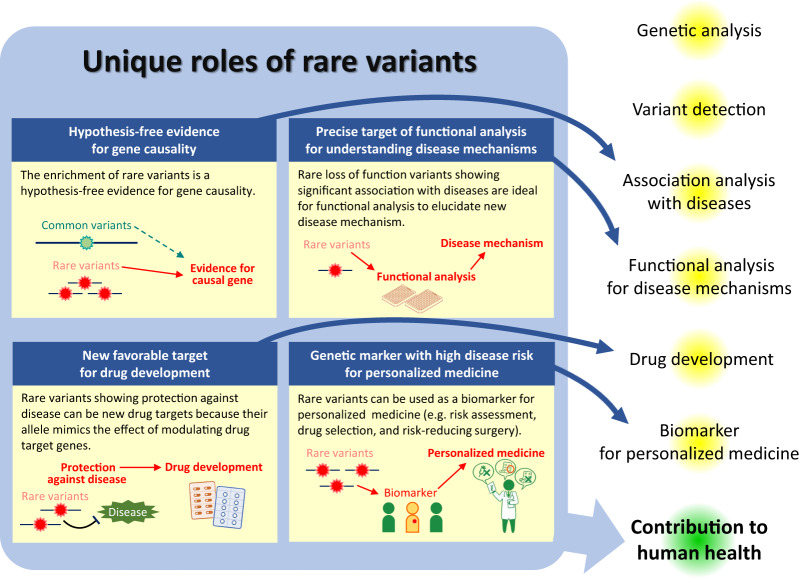
Unique roles of rare variants in the genetic of complex diseases. Rare variants have unique roles which are different from common variants characterized by lower impact on gene function, and higher linkage disequilibrium with flanking variants. In this manuscript, four unique roles are discussed, and they contribute to different parts in the genetics of complex diseases in humans, which ultimately leads to the improvement of human health

In order to analyze the contribution of rare variants to complex diseases, all rare variants will need to be identified by sequencing individuals. Next-generation sequencing (NGS) now allows for whole-genome sequencing (WGS) to be performed for under 1000 dollars [9] and WGS studies have reported on tens of thousands samples [10]. The importance of rare variants is increasing. The role of rare variants in the genetics of complex diseases in humans is not a simple extension of that of common variants—that is, simply for their association with diseases and phenotypes in WGS-based association studies. Rare variants have distinctive features, including lower linkage disequilibrium with flanking variants, a higher impact of some rare variants on gene function and expression, and a larger population specificity, with which rare variants play unique roles in the genetics of complex diseases. In this review, we discuss the roles played by rare variants in the genetics of complex diseases (Fig. 1), including especially inflammatory bowel diseases (IBD) consisting of Crohn’s disease and ulcerative colitis [11], and hereditary cancers the authors have contributed to.

It is worth noting that the threshold of MAF in rare variants has not yet been clearly defined. As shown in Table 1, this threshold varies between 0.1% and 5% in previous studies. The terms “low-frequency variants” and “less common variants” are used to indicate variants whose frequency lies between common variants and rare variants. However, since the unique roles of rare variants described in this review are slightly changed according to the threshold of MAF but generally applicable to any threshold of MAF, they will be defined where necessary.

**Table 1:** Diseases associated with the enrichment of rare variants (sorted in ascending order)

Disease	Gene	Method to identfy variants	Variant selection	MAF	Carrier freq. in cases	Carrier freq. in controls	No of cases	No of controls	*P*	OR (95% CI)	Ref
Rheumatoid arthritis	*IL2RA, IL2RB*	TS	Nonsynonymous	5%	0.8%, 1.0%	0%, 0.2%	500	650	0.007, 0.018	NA	[104]
Age-related macular degeneration	*CFI*	TS	Nonsynonymous	1%	7.8%	2.3%	1676	745	1.6 × 10^−8^	3.57	[105]
Coronary artery disease	*APOC3*	Exome chip	LoF	1%	0.3%	0.5%	33,889	76,583	4 × 10^−6^	0.60 (0.47–0.75)	[106]
Type 2 diabetes	*SLC30A8*	WES, TS, genotyping	LoF and p.Met50Ile	–	0.1%	0.3%	30,433	118,701	1.7 × 10^−6^	0.34 (0.21–0.53)	[107]
Alzheimer’s disease	*ABCA7*	TS	LoF	1%	3.6%	0.1%	772	757	0.0002	RR 4.03 (1.75–9.29)	[108]
Schizophrenia	*SETD1A*	WES	LoF	0.1%	0.2%	0%	4264	9343	0.0003	–	[109]
Age-related macular degeneration	*CFB, CETP*	TS	Nonsynonymous	5%	5.3%, 12.1%	9.2%, 8.1%	2886	9337	4.42 × 10^−11^,4.27 × 10^−11^	0.55 (0.46–0.66), 1.57 (1.37–1.80)	[110]
Early-onset coronary artery disease	*LPL*	WES	LoF, ClinVar pathogenic, and predicted damaging missense	1%	0.6%	0.3%	10,138	12,395	0.0010	1.96 (1.30–2.96)	[111]
Early-onset Atrial fibrillation	*TTN*	WGS	LoF	1%	2.1%	1.1%	2047	2116	0.0342	1.76 (1.04–2.97)	[112]
Idiopathic pulmonary fibrosis	*TERT, PARN, TERC, RTEL1*	WGS	Missense and LoF	1%	8.6%^a^	2.4%^a^	1739	8645	2.44 × 10^−8^	–	[113]
Type 2 diabetes	*ADCY3*	WES	LoF	5%	0.08%	0.01%	8845	9323	0.044	8.6 (1.1–69.5)	[114]
Rheumatoid arthritis	*TYK2*	TS	Nonsynonymous	5%	8.3%	14.0%	2294	4461	3.94 × 10^-12^	0.56 (0.47–0.66)	[32]
Early-onset Alzheimer’s disease, frontotemporal dementia	*TET2*	WGS	Coding and non-coding variants with CADD > 10	Private	4.1%	0.1%	435	671	4.6 × 10^−8^	28.9 (4.5–1200)	[115]

*OR* odds ratio, *LoF* loss of function, *WGS* whole genome sequencing, *WES* whole exome sequencing, *TS* target sequencing, *RR* relative risk, *MAF*minor allele frequency, *CI* confidence interval

^a^Frequency of individuals with rare variants in at least one of four genes

## Unique roles of rare variants

### Hypothesis-free evidence for gene causality

The identification of causal genes responsible for disease onset is one of the goals of biological research. Various types of evidence are used to denote gene causality. Among them, hypothesis-free evidence is obtained by a hypothesis-free approach in which researchers do not start with a certain functional hypothesis [12]. GWAS is considered as a hypothesis-free approach because GWAS systematically analyzes SNPs across genome without a prior functional hypothesis. This type of evidence is considered robust because it does not depend on the accuracy of a prior functional hypothesis. Rare variants could provide hypothesis-free evidence for gene causality in complex diseases.

In 2001, two groups [13, 14] identified three common variants (p.Arg702Trp, p.Gly908Cys, and p.Leu1007ProfsTer2) in *NOD2* that independently increased the risk of Crohn’s diseases [9]. In addition, the French group sequenced the coding regions of *NOD2* in 457 patients with Crohn’s disease and 103 unaffected individuals to identify rare nonsynoymous variants. They found that patients had more rare nonsynonymous variants (17%) than unaffected individuals (5%). These results suggest that not only three common variants, but also rare variants, contribute to an increased risk of Crohn’s disease because linkage disequilibrium between common variants and rare variants is low. Since the identification of enrichment of rare variants is conducted without a prior functional hypothesis, rare variants could provide hypothesis-free evidence for gene causality independent of common variants.

This finding gained further attention around 2008, when GWAS became more widely reported. Several early GWAS in 2006 and 2007 [15–18] typically identified a few SNPs showing genome-wide significant association. Given a low-expected odds ratio (<1.2), large sample sizes were needed to improve the identification of SNPs with genome-wide associations. Three groups combined their GWAS data as a meta-analysis with adjustment for differences in SNP arrays by imputation [19] to identify as many as 32 loci (21 additional loci in this meta-analysis) associated with Crohn’s disease. Meta-analysis for other complex diseases also identified more loci around the same time, including 30 loci for polygenic dyslipidemia [20] and 10 loci for colorectal cancer [21]. Although meta-analysis dramatically increased the number of loci identified, the causal variants and genes remained largely unknown. To identify likely causal genes, various fine-mapping strategies were used, including expression quantitative trait locus (eQTL), functional annotation of genetic variants, PubMed text mining, protein–protein interactions, pathway prioritization protocol, and Bayesian methods to identify credible sets [22]. Among these, the use of Bayesian methods for the identification of credible sets [23] and the identification of rare variant enrichment follow the advantage of the hypothesis-free approach of GWAS.

Two groups from Belgium [24] and the USA [25] tried to identify rare variant enrichment in positional candidate genes (63 and 56 genes, respectively) in IBD using NGS. *IL23R* showed that controls had more rare variants than Crohn’s disease patients [24]. This enrichment was also observed in ulcerative colitis patients, consistent with GWAS results [18]. In addition, single rare coding variants in *CARD9*, *IL18RAP*, *CUL2*, *C1orf106*, *PTPN22*, and *MUC19* showed additional associations [25]. As described in Table 1, the enrichment of rare variants was also investigated in other complex diseases. Whole exome and genome sequencing have also been used for IBD and other complex diseases. Luo et al. identified a novel rare missense variant in *ADCY7* associated with ulcerative colitis [26]. In addition, IBD genes implicated by causal coding or eQTL variants showed the enrichment of rare variants in Crohn’s disease [26]. In the era of WGS, the enrichment of rare variants has been widely investigated (Table 1).

A statistical method about rare variants was also evolved. A higher statistical power was obtained in the above analysis method if all rare nonsynonymous variants were functional with the same magnitude and direction of impact on gene function. However, nonsynoymous variants can be nonfunctional, while others may have opposite effects in terms of function. Researchers also wanted to include various covariates such as sex and age into statistics analysis. As results, various statistical analyses have been proposed to maintain or increase statistical power in these different scenarios, which were grouped into four categories: burden tests, variance-component tests, combined tests, and other tests [27]. Details were reviewed by Povysil [28].

Statistical analysis is conducted in functional units because variants in the same functional unit are expected to show similar functions for the same target. In most cases, the coding regions of one gene have been used as a functional unit, since a target is the same and the annotation of variants, including synonymous, nonsynonymous, and loss-of-function variants, could be reliably determined. Multiple genes have also be investigated in the form of functional units, including prior knowledge associated gene sets [29, 30], sets of candidate genes selected by eQTL [26, 31], and known pathways [32], although some are not hypothesis-free approaches. Gene ontology is also used to characterize genes with variants [33]. Other researchers have focused on specific regions within a single gene. Specific known domains [32] and regions with different missense tolerance ratios [34] are used to identify functionally important parts. In this way, the role of hypothesis-free evidence for gene causality is being evolved.

### Precise target of functional analysis for understanding disease mechanisms

GWAS and other hypothesis-free genetic analysis methods are expected to identify unknown mechanisms that cannot be identified using knowledge-based research. The identification of causal variants is plausible for further functional analysis. However, this is difficult, especially for common variants, since they are generally found in high linkage disequilibrium with flanking variants. In addition, it is common to have multiple causal variants, including rare variants in one GWAS locus. Therefore, it is challenging to identify a causal variant according to the association pattern. For example, the IRGM locus was previously identified in a GWAS on Crohn’s disease, wherein a 20-kb common deletion 2 kbp upstream of *IRGM* was considered to be a likely causal variant due to the fact that it showed perfect linkage disequilibrium with the highest signal and an eQTL effect on *IRGM* [35]. Three years later, a common synonymous variant (c.313C>T) was also considered as a potential causal variant. This variant also showed perfect linkage disequilibrium with the highest signal, but had been disregarded due to lacking an amino acid change. Brest et al. [36] found that microRNA (miR-196) was overexpressed in the inflammatory intestinal epithelia of patients with Crohn’s disease. c.313C>T was located within the seed region of miR-196 with different IRGM expression under the control of miR196.

Similar difficulty was observed in the *FTO* locus of individuals with obesity. While a top variant identified by GWAS was assumed to regulate *FTO* by a series of functional tests in 2009–2010 [37, 38], in 2014 it was found to interact with the promoters of *IRX3* located several hundred kilobases away and obesity-associated variants were associated with *IRX3* expression [39]. Indeed, Irx3-deficient mice showed a reduction in body weight of 25–30% primarily through the loss of fat mass and increase in basal metabolic rate with browning of white adipose tissue. Functional analyses are indispensable for the identification of the mechanisms of variants in disease onset. However, it remains difficult for researchers to validate mechanisms revealed by functional analysis since they are unable to test all possible mechanisms.

A potential strategy to decrease the possibility of this type of misinterpretation is to focus on loss-of-function (LoF) variants, such as nonsense, frame-shift, and splice-site variants, since the ambiguity about the direction and the magnitude of impact on gene function is limited. In particular, rare LoF variants showing significant association with diseases are ideal. *CARD9* in IBD is one such example. While a common GWAS signal in this locus is considered to change the expression level of *CARD9* [31], IVS11+1C>G presumably skipping exon 11 was also identified using target sequencing in candidate genes. The frequencies in cases and controls were 0.20% and 0.64%, respectively (*P* < 1×10^−16^; odds ratio = 0.29) [25]. A subsequent study with this rare LoF variant showed that ubiquitin ligase TRIM62 regulates *CARD9*-medicated anti-fungal immunity and intestinal inflammation [40]. Other examples about functional analysis with rare variants associated with diseases are provided in Table 2.

**Table 2:** Functional assay with rare variants for disease mechanisms

Disease	Gene	Variants for functional assay	Effect of variants	Ref
Autism spectrum disorder	*SLC6A3*	p.Thr356Met	Alterations in dopamine homeostasis mediated by aberrant dopamine transporter function	[116]
Inflammatory bowel disease	*CARD9*	IVS11+1C>G	Ubiquitin ligase TRIM62 regulates CARD9-medicated anti-fungal immunity and intestinal inflammation	[40]
Multiple sclerosis, systemic lupus erythematosus	*TNFSF13B*	Indel in 3′ UTR	Increased production of soluble B cell activating factor by escaping microRNA inhibition leading to up-regulated humoral immunity	[117]
Esophageal squamous cell carcinoma	*CYP26B1*	p.Arg323Trp	Enhanced catabolic activity of CYP26B1 resulting in decrease of serum all-trans retinoic acid	[118]
Pancreatic cancer	*RABL3*	p.Ser36Ter	Accelerated KRAS prenylation resulting in cell proliferation	[119]
Systemic lupus erythematosus	*BLK*	p.Arg131Trp	Impaired suppression of IRF5-mediated type-I interferon expression	[120]
Type 2 diabetes	*ZnT8*	p.Arg138Ter	Increased glucose responsiveness and reduced K_ATP_ channel function	[121]
Parkinson’s disease	*LRRK2*	p.Gly2019Ser	Increased LRRK2 kinase activity	[122, 123]
Idiopathic pulmonary arterial hypertension	*PTGIS*	p.Arg252Q, p.Ala447Thr	Decreased prostacyclin production and increased cell death of pulmonary microvascular endothelial cells	[124]

If researchers were to directly analyze the participants, it would be possible to deeply understand biological mechanisms behind genetic associations. Generally, studies have identified variants associated with phenotypes in previously collected individuals. When participants are recruited to a study, the volume of phenotype analysis is limited due to financial and practical constraints for the optimal number of participants that ensures the best statistical power. However, when a specific potentially causal variant is being studied, recruitment is limited to participants with the genotypes of interest to allow researchers for deep phenotyping, which is called Recall-by-Genotype [41].

One example of the Recall-by-Genotype is *TYK2*. *TYK2* is differentially associated with common autoimmune diseases, including Crohn’s disease, ulcerative colitis, ankylosing spondylitis, multiple sclerosis, and psoriasis [42]. Dendrou et al. tried to resolve genotype-to-phenotype differences in autoimmunity in this locus with a potentially rare causal variant. They performed fine-mapping to identify rs34536443 (p.Pro1104Ala). Among their analyses, they recalled individuals from the Oxford BioBank for blood donation in a balanced, age- and sex-matched fashion based on preselected heterozygotes and homozygotes of a minor allele. In their study, peripheral blood mononuclear cells were separated into CD3^+^ T cells, CD4^+^ T cells, CD8^+^ T cells, CD19^+^ B cells, and CD14^+^ monocytes, followed by cytokine stimulations of peripheral blood mononuclear cells, which are unlikely to be collected without Recall-by-Genotype. The authors found that a potentially causal variant (rs34536443) had a demonstrable impact on *TYK2* function, leading to impaired type I IFN, IL-12, and IL-23 signaling. This indicates that studies analyzing disease mechanisms have a greater chance of elucidating the causal mechanism of disease if they study a specific causal variant in subjects with genotypes of interest.

### New favorable target for drug development

Accumulating information on the associations between genes and diseases is also important for drug development. Drug candidates with genetically supported targets are more likely to be successful in Phase II and III clinical trials [43]. Genetic associations can also be used to identify new favorable drug targets. In particular, rare variants that worked protectively against disease have been used to identify new drug targets because their allele mimics the effect of modulating drug target genes [44, 45]. A notable example is *PCSK9*. In black individuals, there are two rare nonsense variants (p.Tyr142Ter and p.Cys679Ter) in *PCSK9*, with 2.6% of individuals having at least one nonsense variant [46]. In 2006, a study on a cohort of black individuals found that nonsense mutations in *PCSK9* resulted in a reduction in the mean LDL cholesterol and the risk of coronary heart disease of 28% and 88%, respectively [46]. In 2017, a monoclonal antibody inhibiting *PCSK9*, evolocumab, reduced the LDL cholesterol levels in individuals receiving statin therapy and the risk of cardiovascular events in a randomized, double-blind, placebo-controlled trial [47]. This drug was subsequently approved and launched.

Along with the development of genetic analysis techniques, methods used for the identification of rare variants that provide protection against disease have evolved. Currently, researchers use biobanks to target 18,228 LoF and 135 phenotypes at one time to systematically identify 27 associations, showing the value of collecting population-scale genomic data [48]. Another idea involves focusing on consanguineous unions, as they are more likely to result in offspring carrying homozygous LoF. In the Pakistan Risk of Myocardial Infarction Study, researchers sequenced the protein-coding regions in 10,503 adult participants to identify 49,138 rare LoF variants. They systematically identified their impact on >200 biochemical and disease traits, especially in homozygotes of LoF [49]. Another group sequenced the exomes of 3222 British adults of Pakistani heritage and identified 1111 rare-variant homozygous genotypes with LoF in 781 genes, although no significant relationship between gene knockouts and clinical consultation or prescription rate was found [50]. While such large-scale screening is promising for identifying new targets for drug development, more traditional strategies, including series of studies performing gene identification, functional analysis, and mechanisms identification, have also resulted in drug development, such as *IL23R* for Risankizumab [51] (Table 3).

**Table 3:** Drugs for complex diseases developed or under development based on the association between rare variants and complex diseases

Disease	Gene	Drug	Ref
Acute coronary syndromes	*NPC1L1*	Ezetimibe (NPC1L1 inhibitor)	[125]
Asthma	*CRTH2*	OC000459 (CRTH2 antagonist)	[126]
Breast and ovarian cancer	*BRCA1/2*	Olaparib (poly (ADP-ribose) polymerase inhibitor)	[127]
Crohn’s disease	*IL23R*	Risankizumab (IL23 inhibitor)	[51]
Erythromelalgia	*SCN9A*	Funapide (Nav1.7 blocker)	[128]
Hypercholesterolaemia	*PCSK9*	Alirocumab, Evolocumab (PCSK9 inhibitor)	[129]
Osteoporosis	*CTSK, SOST*	Odanacatib (cathepsin K inhibitor), Romosozumab (sclerostin antibody)	[130]

### Genetic marker with high disease risk for personalized medicine

Most odds ratios of common variants identified by GWAS are <1.2 in complex diseases. Therefore, most single variants associated with complex disease are not clinically useful, but some complex diseases are caused by a single pathogenic variants in some genes, including *BRCA1* and *BRCA2* in hereditary breast cancer and ovarian cancer syndrome [52], *MLH1*, *MSH2*, *MSH6*, and *PMS2* in Lynch syndrome [53], and *LDLR* and *PCSK9* in atherosclerotic cardiovascular disease [54]. In an individual with a pathogenic variant of *BRCA1*, the cumulative risk for breast cancer and ovarian cancer at 80 years of age is 72% and 44%, respectively [55]. These individuals are expected to have a longer median progression-free survival and a lower risk of disease progression or death by treatment with a PARP inhibitor [56]. Interventions such as risk-reducing bilateral mastectomy, salpingo-oophorectomy, and breast magnetic resonance imaging for early detection could be applied to carriers of pathogenic variants [57]. The same benefits could be obtained by their relatives. Therefore, rare variants have great potential for use as biomarkers in personalized medicine.

This seems a typical example of well-established personalized medicine. However, rare variants have many challenges in order for them to work well. In addition, there are also more potentials in this field. The greatest challenge in this field is the annotation of variants. Recently, a multiple gene panel for the analysis of several genes in one genetic test was used in-clinic to identify genetic variants in a patient. For breast cancer screening, 11 genes, including *BRCA1* and *BRCA2*, are recommended for genetic testing in the National Comprehensive Cancer Network guidelines [57]. Typically, ~10 genetic variants are identified on average in one individual [58] but a subject wants to know whether she has a pathogenic variant that increases risk among the variants detected; this is not easy. If the variant is a LoF variant, in most cases it would be a pathogenic variant. However, this is not always true. The insertion of 4-bp frameshift (p.Lys1358fs, rs55740729) in *MSH6* is known to be a benign variant in Lynch syndrome. Indeed, this variant was equally observed in colorectal cancer patients (2.13%) and controls (2.09%) in a Japanese cohort [59]. In contrast, synonymous variants are likely benign because they do not undergo changes in their amino acid sequences. However, p.Gln1395Gln in *BRCA1* is registered as pathogenic in ClinVar [60] since it alters splicing [61]. Nonsynonymous variants are less straightforward because it is difficult to estimate the direction and magnitude of the impact of each nonsynonymous variant on gene function. To resolve this, guidelines have been developed that allow determining the clinical interpretation of variants. The guidelines by the American College of Medical Genetics and Genomics and the Association for Molecular Pathology have 16 criteria for pathogenicity and 12 criteria for benign. These criteria have to be checked for each variant to determine the appropriate clinical interpretation [57]. Despite this guideline providing a consensus in the community of genetic research, inconsistencies in clinical interpretation have been found between different laboratories [62], and recommendations for the modification of the criteria regarding LoF variants have been discussed [63].

Another difficulty is the differences in pathogenic variants between populations. Most pathogenic variants are singleton variants. For example, 75.8% of 244 pathogenic variants in 11 genes found in breast cancer in Japanese individuals were singleton [58] but there are founder mutations shared in patients. Since founder mutations are specific to racial, ethnic, or geographic groups [64], they change the importance of genes in such groups. For example, three founder mutations in *BRCA1/2* have a combined prevalence of as high as 2–3% in American Ashkenazi Jews [65], while this prevalence is estimated to be 0.1–0.5% in other populations based on Exome Aggregation Consortium data [66]. Other genes were also influenced by population-specific rare variants. In European populations, c.1100delC in *CHEK2*, one of the 11 genes recommended for genetic testing, is common to breast cancer patients in the UK (1.2–1.3%), Netherlands (2.5–3.8%), Finland (2.1–2.9%), Germany (0.33–1.1%), and Australia (0.68%) [67]. On the other hand, this founder mutation has not been observed in Japanese individuals, resulting in the number of patients with pathogenic variants in *CHEK2* in European populations (1.12% of breast cancer patients) to be threefold higher than in the Japanese population (0.38%) [58]. As another example, *NBN* was recommended for genetic testing as a founder mutation (c.657del5, p.Lys219Asnfs, rs587776650) in *NBN* associated with breast cancer [68] and prostate cancer [69] in European populations. However, this variant was not found in Japanese breast or prostate cancer patients, and there were no associations between other pathogenic variants in *NBN* and both cancers in Japanese cohorts [58, 70]. Therefore, *NBN* does not need to be included in Japanese cohorts. The inverse may also be true: Japanese-specific founder mutations in one unknown gene may contribute to increased risk of certain cancers in Japanese. However, there are limited efforts to identify such variants [71, 72].

Despite the various obstacles, the use of variants as biomarkers in personalized medicine has more great potential. Although most research focuses on specific genes and cancers, including *BRCA1/2* for breast and ovarian cancer and *MLH1*, *MSH2*, and *MSH6* for colorectal cancer, more recently, other genes and diseases have been investigated due to the expansion of multi-gene panels. For instance, in as late as 2018, 5.5% of pancreatic cancer patients were found to have germline pathogenic variants in *BRCA1/2*, as well as *ATM*, *CDKN2A*, *TP53*, and *MLH1* (odds ratio = 2.6–12.3) [73]. These proportions and odds ratio are comparable to those of breast cancer. Among metastatic pancreatic cancer patients with germline pathogenic variants in *BRCA1/2*, progression-free survival was reported longer after treatment with a PARP inhibitor in 2019 [74]. Therefore, since other cancers and genes would have such possibilities for personalize medicine with rare variants, further investigations are needed.

Population screening for large-scale precision prevention is another potential approach [75]. Although the ethical, social, and legal implications should be carefully considered, several population screening studies have already been conducted mainly in specific high-risk populations, including Ashkenazi Jews. Even a clinical trial of genomic screening of newborn babies is being running [76]. How these new techniques are introduced into society will require careful consideration. Data accumulation is indispensable for this consideration.

## Future perspectives

As described above, rare variants play unique roles in the genetics of complex diseases. It is worth noting that the four roles described above are not mutually exclusive. Rare variants in *BRCA1/2* played all roles, although the third role was different. A deeper understanding of the function of *BRCA1/2* has led to the development of a new concept, denoted as synthetic lethality [77]. Since NGS will continue to be used to identify new rare variants, these unique roles are likely to become more important. However, several barriers remain to be overcome.

### Sample size

Rare variants require much larger sample sizes than common variants to obtain a sufficiently high statistical power. For instance, when the effect size of a variant is 0.1 (corresponding to an odds ratio of ~1.2) phenotyping standard deviation units, a common variant with MAF = 10% needs ~10,000 individuals to obtain genome-wide significance at *P* = 5 × 10^−8^ with 80% statistical power. MAF of variant = 1% and 0.1% requires ~100,000 and 1 million individuals, respectively. Despite improvement ideas in sample collection [78], genome-sequencing methods [79], and data analysis [80], achieving such large volumes remains a challenge. To overcome this, various methods are used, including statistical analysis [28], imputation [7], target sequencing, and the use of other populations.

As described above, several methods to determine the effects of rare variants in different scenarios have been developed to increase statistical power. However, more care is needed in rare variants than common variants. Association analysis with rare variants is influenced by various factors, including the geographical area of the samples, the timing of sampling, sequencing coverage, and the selection of qualifying variants [28]. Among these, the timing of DNA sampling requires further explanation. One reason is due to age-associated somatic variants in myeloid cancer-associated genes, such as *DNMT3A*, *TET2*, *ASXL1*, and *TP53* in DNA extracted from blood [81]. The prevalence of somatic variants increases with age, from 0% in individuals in their 20 s to 29.4% in individuals over 100 years old. Therefore, somatic *TP53* variants frequently confound genetic testing results, although they are intended to analyze germline variants [82]. Another example is treatment-induced somatic variants. Chemotherapy-induced somatic variants in *PPM1D*, which caused pseudo-associations between breast and ovarian cancers, and variants in *PPM1D* because patients received chemotherapy to induce somatic variants in *PPM1D* [83]. Therefore, the statistical analysis of rare variants requires more care from sampling.

Imputation has evolved by increasing the number of samples and variants, as well as including various populations. Recent public reference panels include UK10K projects (3781 samples, 42.0 million variants, European), Haplotype Reference Consortium (32,470 samples, 40.4 million variants, predominantly European), and Trans-Omics for Precision Medicine (60,039 samples, 239.7 million variants, multiethnic) [84]. An important resource is a website used to perform imputation [8]. Imputation is a computer-intensive task, and not all researchers have access to the servers needed for imputation. Thanks to this website, the number of variants identified from SNP arrays can be increased to enable the analysis of rare variants by imputation.

Target sequencing is used to sequence specific regions of interest based on prior knowledge. The target sequencing of functionally candidate genes was frequently conducted in the 1990s and 2000s before GWAS, although it has been criticized for its low replication rate [85, 86]. However, when focusing on positional candidate genes located in GWAS loci, the rate improves, most likely due to the selection of genes by GWAS increasing the possibility that a target gene is causal for disease. Indeed, the target sequencing of candidate genes identified by GWAS has led to the identification of rare variants associated with diseases with reasonable *p* values (Table 1). Target sequencing could be used to analyze rare variants in a much greater number of samples than WGS and whole-exome sequencing, and thereby reveal the contribution of rare variants with better statistical power.

Another possibility is to use other populations. Even if a certain variant in one gene needs a huge number of individuals to obtain significant associations between variants in one gene and diseases in one population, another population might have a higher frequency of such a variant or more frequent variants with similar functional impact in the same gene to have better statistical power. A typical example is the association between a LoF variant (p.Arg684Ter) in *TBC1D4* and type 2 diabetes in Greenland [87]. The allele frequency of this variant was 17% in this population, but 0.003% in other European populations. Therefore, this variant was not identified to be associated with type 2 diabetes and related phenotypes, most probably because p.Arg684Ter was neither genotyped nor imputed in previous GWAS using European populations. For the same disease, GWAS in Japanese populations identified *GLP1R*, which was previously missed due to lower frequency of variants in European populations [88]. Using the same concept, GWAS with multi-ethnic and admixed populations was conducted to show substantial benefits for fine-mapping and insight on clinical implications [89]. In addition, population specific custom SNP arrays have been used, including Infinium Asian Screening Array, Axiom Japonica Array, and Infinium Multi-Ethnic AMR/AFR. They include population-specific rare variants. These efforts will also compensate for inequitable access to precision medicine in minority populations with a disproportionately higher burden of chronic conditions.

### Estimation of the functional impact of a rare variant

In all four roles of rare variants, it is always important to select functional causal variants. If nonfunctional variants are included, statistical power decreases and functional analysis may be in the wrong direction. Even LoF requires filtering and manual curation for removing artefacts not to dilute association signals [90, 91]. For nonsynonymous variants, several in silico programs, such as SIFT, PolyPhen, Condel, and CADD, provide an estimation of the impact of each variant. However, the resulting estimation of each variant may not be sufficiently accurate [92]. Functional assays have been developed to estimate the functional impact of rare variants. Nonsynonymous variants in *BRCA1/2* have been frequently assayed, focusing on the homology-directed DNA repair function, embryonic stem cell viability, transcriptional activation, drug-sensitivity, protein–protein interaction, and splicing [93], as their functional impact is directly linked to changes in clinical management. However, these efforts have not been successful in decreasing variants with unknown functional impacts due to the fact that functional assays focus on specific functions in *BRCA1/2*, which do not mimic full function in vivo [93]. However, the accurate classification of variants in *BRCA1* using saturation genome editing has been recently reported [93]. In this study, the functional impact of ~4000 variants was assessed, and was almost perfectly concordant with the established clinical interpretation of pathogenicity in ClinVar. A similar strategy could be applied to other genes, although the experimental condition needs optimization according to the genes being studied. In addition, while the clinical interpretation of a large number of variants in *BRCA1/2* has been already submitted to ClinVar [60] and could be used as “ground truth positive” to optimize experiments, a limited number of variants in other genes have been deposited. Therefore, the sharing of clinical interpretation data on variants in other genes is indispensable for the development of high-throughput functional assays in other genes.

### Beyond coding regions

The unique roles of rare variants have been mainly played in coding regions. However, there is evidence that rare variants in non-coding regions have a large impact on gene expression and disease. A large deletion at the 3′-end of *EPCAM* is known to cause allele-specific epigenetic silencing of the neighboring DNA mismatch repair gene *MSH2*, leading to Lynch syndrome [94]. Hernandez et al. reported that singletons contribute to ~25% of cis eQTL heritability across genes [95]. A variant in the 5′ untranslated region that is known to result in the methylation-associated silencing of *BRCA1* is dominantly inherited in some families affected by breast and ovarian cancer [96]. The association between genetic variants and multi-omics data, including transcriptome, post-transcriptional regulation, epigenome, protein post-translation modification, metabolome, and microbiome data, has helped to improve our understanding of rare variants in non-coding regions [97]. However, estimating the impact of rare variants on target genes at the single-base resolution remains a challenge. Nevertheless, a new model with a novel experimental approach, CRISPRi-FlowFISH, has been proposed for interpreting the functions of variants in non-coding regions [98]. In addition, various in silico prediction tools for non-coding regions are being developed, including regBase [99], RegSNPs-intron [100], and GRAM [101]. Overall, a better understanding of variants in coding and non-coding regions and single variant annotation across whole genome would take advantage of population-based sequencing data to provide great benefits to human health.

### Integration of genetic and non-genetic information

One of the goals in the characterization of variants is to provide diagnosis and forecast of future disease risk. In this context, researchers will have to consider all genetic variants across the entire genome, including structural variants such as copy number variations, insertion, inversions, and translocations [102]. The functional impact of each variant discussed above should also be included in the calculation. In addition, electronic health record including digital image, data from health monitoring device, and other environmental exposure might be considered alongside. Artificial intelligence is expected to deal with all the information [103]. However, there are various challenges and limitations, including regulatory issues, interpretability, and data and machine bias. Therefore, large-scale training and validation datasets about genomics, electronic health record and other information are needed for artificial intelligence to integrate genetic and non-genetic information to provide diagnosis and forecast of future disease risk.

## Conclusion

Rare variants play unique roles in the genetics of complex diseases in humans, including as hypothesis-free evidence of gene causality, a precise target of functional analysis for understanding disease mechanisms, a new target for drug development, and a genetic marker with high disease risk for personalized medicine (Fig. 1). Advances in WGS will continue to allow for the identification of rare variants, where a better estimation of the functional impact of each rare variant across the whole genome will provide paramount benefits to human health.
